# Predictive Role of Pre-Operative Anemia in Early Recurrence of Endometrial Cancer: A Single-Center Study in Romania

**DOI:** 10.3390/jcm13030794

**Published:** 2024-01-30

**Authors:** Mihaela Ionică, Marius Biris, Florin Gorun, Nicoleta Nicolae, Zoran Laurentiu Popa, Maria Cezara Muresan, Marius Forga, Dragos Erdelean, Izabella Erdelean, Mihai Adrian Gorun, Octavian Constantin Neagoe

**Affiliations:** 1 Second Clinic of General Surgery and Surgical Oncology, Emergency Clinical Municipal Hospital, 300079 Timisoara, Romania; ionica.mihaela@umft.ro (M.I.); neagoe.octavian@umft.ro (O.C.N.); 2 Second Discipline of Surgical Semiology, First Department of Surgery, “Victor Babes” University of Medicine and Pharmacy, 300041 Timisoara, Romania; 3 Department of Obstetrics and Gynecology, “Victor Babes” University of Medicine and Pharmacy Timisoara, 300041 Timisoara, Romania; nicolae.nicoleta@umft.ro (N.N.); popa.zoran@umft.ro (Z.L.P.); muresan.maria@umft.ro (M.C.M.); forga.marius@umft.ro (M.F.); erdelean.dragos@umft.ro (D.E.); erdelean.izabella@umft.ro (I.E.); 4 Department of Obstetrics and Gynecology, Municipal Emergency Clinical Hospital Timisoara, 300172 Timisoara, Romania; gorun.florin@umft.ro; 5 Oncohelp Oncology Center, 300239 Timisoara, Romania; gorun.adrianm@yahoo.com

**Keywords:** anemia, endometrial cancer, oncologic surgery

## Abstract

This study aims to investigate the association between anemia and early recurrence in endometrial cancer patients. We retrospectively analyzed the data of 473 endometrial cancer patients treated at our hospital from January 2015 to December 2020. Patients were divided into two groups based on their hemoglobin (Hb) level: anemia group (Hb < 12 g/dL) and non-anemia group (Hb ≥12 g/dL). Early recurrence was defined as recurrence within 2 years of diagnosis. Univariate and multivariate logistic regression analyses were used to identify the predictors of early recurrence. The prevalence of anemia was 38.26% (181/473). The incidence of early recurrence was 12.89% (61/473) in the anemia group and 9.24% (38/412) in the non-anemia group (*p* = 0.004). Univariate analysis showed that anemia was a significant predictor of early recurrence (odds ratio (OR) = 2.27, 95% confidence interval (CI): 1.35–3.80, *p* = 0.003). Multivariate analysis confirmed that anemia was an independent predictor of early recurrence (OR = 2.11, 95% CI: 1.21–3.84, *p* = 0.01). Anemia is an independent predictor of early recurrence in endometrial cancer patients. Patients with endometrial cancer should be screened for anemia and treated if present. Additionally, patients with anemia should be closely monitored for early signs of recurrence and treated aggressively.

## 1. Introduction

Endometrial cancer (EC) stands as the most prevalent type of gynecological cancer in Europe and the United States (US) [1,2]. Furthermore, with over 400,000 new cases reported annually, EC ranks as the the sixth most common cancer among women globally, with both incidence and mortality rates on the rise [3,4]. According to projections from the American Cancer Society, an estimated 66,200 new cases of uterine cancer may have been diagnosed in the U.S., with nearly 13,030 women dying by 2023. However, these estimates also include uterine sarcomas which account for up to 10% of cancers of the uterine body, so the real figures for endometrial cancers and deaths are slightly lower than these projections [5].

In most cases, endometrial cancer is associated with a favorable prognosis and high survival rates, due to early diagnosis and good response to standard treatment. The EUROCARE-5 research revealed that among European women diagnosed with endometrial cancer between 2000 and 2007, the 5-year relative survival rate stood at 76%, ranging from 72.9% in Eastern Europe to 83.2% in Northern Europe [6].

Recurrent disease can be seen in 10% up to 15% of patients, the clear majority of cases occurring within three years from the initial diagnosis [7,8]. Numerous tumor and patient characteristics have been suggested as prognostic factors for the recurrence of endometrial cancer, among which age, International Federation of Gynecology and Obstetrics (FIGO) stage, histopathological tumor grade, DNA ploidy, lymph-vascular space invasion have shown the strongest correlation with relapse of disease [8,9,10,11].

The presence of anemia before the initiation of any type of therapy has also been suggested to have a negative influence on patient outcome and survival, but its predictive role has not been clearly described [12,13,14,15,16,17,18]. Also, in patients with endometrial cancer, the prevalence of preoperative anemia treatment is high, 26.5%, according to a systematic review [17]. Patients with a hemoglobin level ≤ 11 g/dL should be evaluated as soon as possible for potential causes of anemia, including iron, nutritional, and hemolysis studies, according to the NCCN Clinical Practice Guidelines in Oncology (NCCN Guidelines) for Hematopoietic Growth Factors [19]. Erythropoiesis-stimulating agents (ESAs), intravenous (IV) or oral iron preparations, red blood cell (RBC) transfusions, and combinations of these therapies are used to treat anemia associated with cancer [20,21]. On the other hand, additional study indicates that red cell transfusions have a negative impact on endometrial cancer patients’ prognosis [22].

Some studies have also shown a link between preoperative anemia and surgical difficulties in colon cancer patients, and optimising hemoglobin levels preoperatively may lead to better outcomes [23]. However, international guidelines on endometrial cancer do not present standardised methods of screening and preoperative treatment of anemia.

Cancer-related death rates have recorded a continuous decline in the past decade therefore it is important to identify negative prognostic factors as to properly asses and elaborate corresponding treatment protocols to maintain this trend and to improve patient survival [1,24,25].

This study aims to examine the role of pre-treatment anemia in patients with endometrial cancer as a predictor of recurrence. It also aims to determine the predictive role of anemia in the mortality rate of endometrial cancer. In this study, we intend to make a significant contribution to the existing literature by exploring, for the first time in Romania, the pivotal role of preoperative anemia in mortality and early recurrence among patients with endometrial cancer. Our research, comprising one of the largest study sizes in the current literature on this specific topic, as can be seen by looking at the study size of studies included in a thorough systematic review [17].

Based on existing literature observations, we hypothesize that the presence of preoperative anemia may serve as an independent predictor of both increased mortality and early recurrence in patients with endometrial cancer.

## 2. Materials and Methods

### 2.1. Study Design and Setting

This retrospective cohort study was conducted on 437 consecutive women who underwent surgery at the Second Clinic of General Surgery and Surgical Oncology, Municipal Emergency Clinical Hospital, Timisoara, Romania, between January 2015 and December 2020. The patients were not diagnosed in our clinic, being admitted to our department with complete diagnosis and staging for surgical treatment.

The study was approved by the Ethics Committeee of the Municipal Emergency Clinical Hospital Timisoara (no. 5/11 November 2014).

This study was conducted in accordance with the Strengthening the Reporting of Observational Studies in Epidemiology (STROBE) guidelines for cohort studies.

### 2.2. Participants

Eligibility criteria included women with histologically confirmed endometrial cancer who underwent surgery at the specified clinic. Exclusions were made for those with a history of endometrial cancer, other malignancies, or incomplete data. After discharge, a comprehensive follow-up protocol was implemented to monitor the health status of participants and to assess for recurrence. The follow-up period extended for a maximum of 5 years post-surgery. Patients were scheduled for regular follow-up appointments at specified intervals, including the first month, at three months, at six months, and then annually.

### 2.3. Variables

The primary outcome was early recurrence, defined as recurrence within 2 years of surgery. The secondary outcome was the mortality. Disease-free survival was defined as the time elapsed from the date of primary surgery for endometrial cancer to the date of disease recurrence or the last follow-up if no recurrence was observed. On the other hand, Overall Survival (OS) was defined as the time from the date of primary surgery to the date of death or the last follow-up if the patient was still alive.

The independent variable was anemia. Potential confounders included age, FIGO stage, lymph node metastasis, adnexal involvement, cervical involvement, myometrial invasion > 50%, and non-endometroid histology, and lymphovascular invasion.

### 2.4. Data Sources/Measurement

Data on anemia, age, FIGO stage, lymph node metastasis, and early recurrence were collected from electronic medical records. Data were verified for accuracy and completeness. Anemia was defined as a value of hemoglobin lower than 12 g/dL.

### 2.5. Statistical Methods

Statistical analysis was performed using Python and RStudio. Continuous variables were presented as median and interquartile range (IQR) and compared using the Mann-Whitney-U test. The normality of the distribution of continuous variables was tested using the Shapiro-Wilk test. Categorical variables were presented as absolute counts and percentages and compared using Fisher’s exact test.

Kaplan-Meier estimates were used for survival analysis, and the log-rank test was applied to compare survival curves.

Cox-regression models were used for the evaluation of correlation between pretreatment and clinicopathological characteristics and disease-free survival. Regression coefficients (Coef.), hazard ratios (HR), standard errors (SE), and *p*-values were calculated to quantify the strength and significance of these associations.

Univariate and multivariate logistic regression analyses were used to identify the predictors of early recurrence and mortality. This analysis provided odds ratios (OR) with corresponding standard errors (SE) and *p*-values, providing information on the probability of death associated with the presence of anemia in both univariate and multivariate contexts.

Statistical significance was set at *p* < 0.05.

## 3. Results

### 3.1. Clinical Characteristics

The study encompassed a cohort of 473 participants, with a median age of 58 years. Predominantly, the cohort presented with a diagnosis of endometrioid carcinoma (82.87%). Staging, as per the FIGO, revealed a majority of patients being situated at stage II during the time of surgery (42.7%). Lymph node metastases were identified in only 10.35% of the participants. The main therapeutic intervention performed in the cohort was hysterectomy (HTV), with a rate recorded in 97.46% of patients.

The prevalence of anemia was 38.26% (181/473). Patients in the anemia group were older (58 years vs. 57 years, *p* = 0.13) and had a higher prevalence of advanced FIGO stage (IIIA-IVA) (41.95% vs. 22.96%, *p* < 0.0001), myometrial invasion (86.49% vs. 79.79%, *p* < 0.0001), lymph node metastases (10.35% vs. 7.53%, *p* = 0.01), adnexal involvement (30.86% vs. 24.31%, *p* = 0.0001), cervical involvement (66.80% vs. 64.38%, *p* = 0.16), and lymphovascular invasion (91.54% vs. 86.98%, *p* < 0.0001). The recurrence rate was higher in the anemia group than in the non-anemia group (12.89% vs. 9.24%, *p* = 0.004) (Table 1).

### 3.2. Survival Analysis

In an attempt to understand the influence of anemia on survival after surgery in patients with endometrial cancer, a Kaplan-Meier survival analysis was performed. In the cohort without pre-surgical anemia, a median survival of 157 months (SE = 5.12) is reported. However, it is important to note that the upper limit of the 95% confidence interval remains undetermined and the lower bound is 132. In contrast, in the anaemia group, the analysis indicates a median survival of 72 months (SE = 4.19). The 95% confidence interval for this cohort shows a lower bound of 66 and an upper bound of 83.

**Figure 1:**
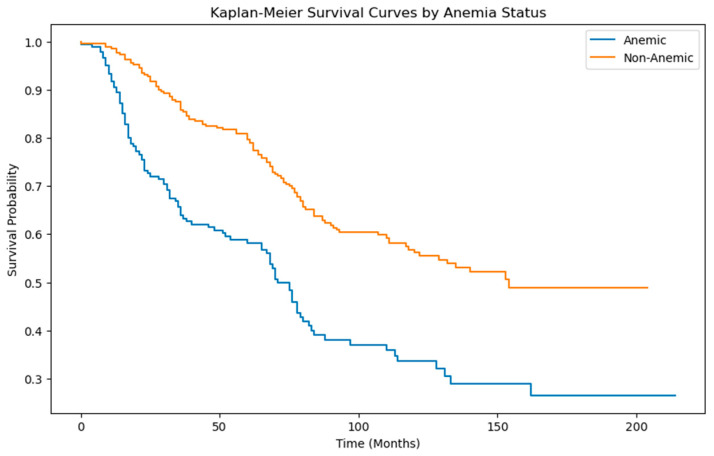
Kaplan-Meier curve of overall survival in endometrial cancer patients by anemia status.

The survival distributions of the two groups are statistically different, the log-rank test statistic of 31.73 is statistically significant, with a *p*-value < 0.0001 (Figure 1).

**Figure 2:**
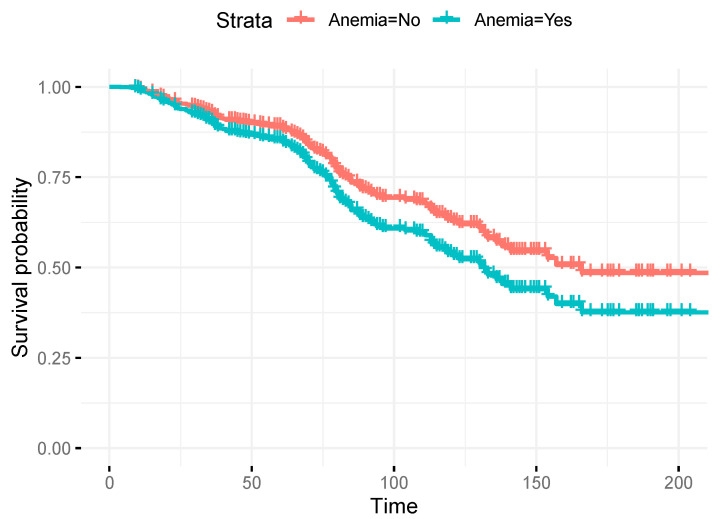
Cox regression survival curve comparing pre-surgery anemia with those who are not anemic.

Cox regression analysis allowed adjustment for potential confounders such as patient age, FIGO stage and lymph node metastases. Also, the adjusted the survival rate of endometrial cancer patients with anemia is lower than that of patients without anemia (Figure 2).

Moreover, the application of Cox proportional hazards regression analysis has elucidated compelling insights into the prognostic determinants of overall survival. Noteworthy among these factors are anemia, age, FIGO stage, and the presence of lymph node metastasis, all of which have exhibited statistical significance in influencing the overall survival outcomes (Table 2).

To expound upon the observed associations, the hazard ratio (HR) for anemia manifests as 1.36, denoting a 36% elevated likelihood of mortality among patients afflicted with anemia in comparison to their non-anemic counterparts. The age-related HR stands at 1.08, implying that with each successive year of age, the risk of mortality escalates by 8%. Furthermore, the HR for FIGO stage III registers at 4.06, signifying a 4.06-fold augmented risk of mortality for patients diagnosed with FIGO stage III disease relative to those with FIGO stage I disease. This risk amplification is notably more pronounced in FIGO stage IV disease, with an HR of 18.94, indicating a substantial 18.94-fold increase in the likelihood of mortality compared to FIGO stage I counterparts. The HR for lymph node metastasis is computed as 2.03, elucidating a 103% heightened probability of mortality for patients with lymph node metastasis in contrast to their counterparts without such metastatic involvement. Finally, HR of myometrial invasion greater than 50% is 1.04, with no statistical difference compared to those without myometrial invasion/ invasion <50%. Also, cases with non-endometroid histology have an HR for mortality of 1.50 compared to cases with endometroid histology (Table 2).

According to binomial logistic regression, the presence of anemia had an OR of 2.14 (*p* < 0.0001) for death in univariate analysis and 1.60 (*p* = 0.04) in multivariate analysis (Table 3).

### 3.3. Anemia as a Predictor for Early Recurrence

In univariate analysis, anemia was associated with a 2.27-fold increased risk of early recurrence (OR = 2.27, *p* = 0.003). Upon subjecting the data to multivariate scrutiny, anemia has persisted as a discernible and statistically significant harbinger of early recurrence, as reflected by an Odds Ratio (OR) of 2.11 (*p* = 0.01) (Table 4).

**Figure 3:**
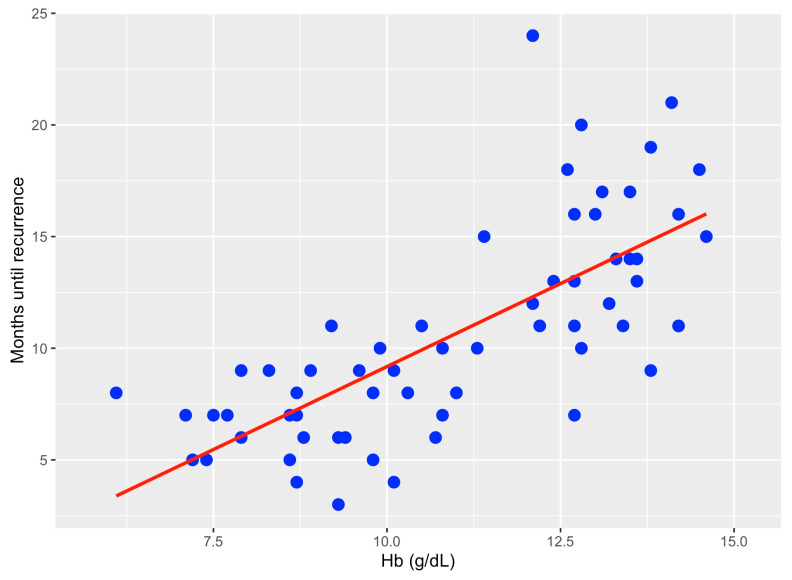
Correlation between Hb count (g/dL) and time in months to endometrial cancer recurrence.

In addition, Spearman correlation analysis shows a strong positive monotonic correlation between time of recurrence in months and hemoglobin value (rho = 0.762; *p* < 0.0001) (Figure 3).

Moreover, individuals afflicted with anemia exhibited a notably elevated hazard ratio (HR) of 11.64 for the occurrence of recurrence, denoting an 11.64-fold increased likelihood compared to their non-anemic counterparts (*p* < 0.0001). Additionally, with each successive year of age, there was an appreciable escalation in the risk of recurrence, with a hazard ratio (HR) of 1.044 and a corresponding *p*-value of 0.006. This implies a 4.4% increment in the hazard of recurrence for each additional year of age, thereby accentuating age as a discernible contributor to the recurrence risk profile. Furthermore, patients harboring lymph node metastasis displayed a hazard ratio (HR) of 2.37 for recurrence, signifying a 2.37-fold heightened likelihood of experiencing recurrence relative to their counterparts devoid of lymph node metastasis (*p* = 0.04) (Table 5).

**Figure 4:**
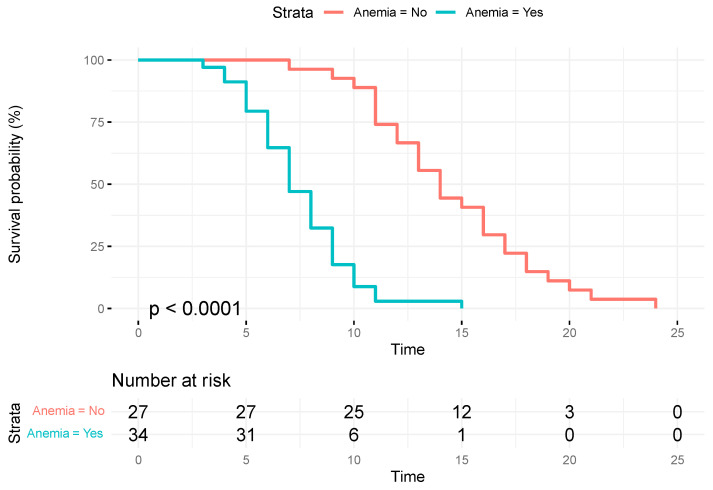
Kaplan-Meier curve of overall time until recurrence by pre-surgery anemia status.

Kaplan Meier analysis shows that in the cohort without preoperative anaemia, a median time to recurrence of 14 months (95%CI = 13–17) is reported compared to the group with anaemia, which had a median time to recurrence of 7 months (95%CI= 7–9). The difference is statistically significant (*p* < 0.0001) (Figure 4).

## 4. Discussion

The study found that pre-operative anemia significantly impacts the prognosis of early recurrence in endometrial cancer. In a cohort of 473 women with endometrial cancer, 38.26% had anemia. Anemic individuals had a higher recurrence rate (12.89% vs. 9.24%). Anemia is a strong predictor of recurrence, with anemic patients showing a recurrence rate exceeding twofold in both univariate and multivariate analyses. In a COX regression analysis, pre-operative anemic patients had a hazard ratio of 11.64 for recurrence. This highlights the heightened susceptibility to recurrence associated with anemia, even when accounting for other clinical factors. Survival analysis, employing the log-rank test, substantiated a compelling and statistically significant distinction in survival distributions between the two aforementioned groups (*p* < 0.0001). This discernible difference underscores the substantive impact of pre-operative anemia on the overall survival outcomes within the studied population.

The Cox regression analysis identified anemia, age, FIGO stage, and lymph node metastasis as significant prognostic factors for overall survival in endometrial cancer. This comprehensive approach emphasizes the clinical importance of pre-operative anemia in endometrial cancer prognosis and its significance in clinical decision-making. Anemia in cancer patients is a common finding at diagnosis, that can be seen in both solid and hematologic malignancies. The presence of an abnormal red blood cell count varies largely depending on the type of primary cancer [26,27]. Among gynecological malignancies, the prevalence of anemia in endometrial cancer varies largely [13,14]. An exact cause for cancer-related anemia has not been “pinpointed” but multiple pathophysiological mechanisms have been suggested: increased inflammatory cytokine production that may lead to a lowering of red blood cell number through either erythropoietin synthesis inhibition or by blocking the maturation of erythroid progenitor cells. Other suggested causes include bone marrow infiltration through malignant, amyloid or other types of deposits, iron deficiency due to inefficient use or reduced nutritional intake, and hemorrhage comprising intratumoral bleeding and, in the case of endometrial cancer, vaginal bleeding episodes.

Hematologic and biological characteristics of cancer-related anemia are similar to those of anemia linked to chronic inflammatory diseases. The production of pro-inflammatory cytokines, primarily IL-6, by immune cells and tumor cells is a significant factor in the etiopathogenesis of anemia linked to cancer. They stimulate alterations in erythroid progenitor proliferation, erythropoietin (EPO) synthesis, survival of circulating erythrocytes, iron balance, redox state and energy metabolism, all of which can contribute to anemia [28]. According to Adamson, there are several pathogenetic mechanisms through which inflammation can lead to anemia, including: (1) Reduced erythropoiesis in bone marrow; (2) Effects of inflammation on erythropoietin production; (3) Changes in iron metabolism that lead to iron-restricted erythropoiesis induced by hepcidin increase [29].

Furthermore, anemia is linked to diminished oxygen transport to the tumor microenvironment; this indicates negative prognostic tumor hypoxia. A chronic cancer-related anemic microenvironment may foster toxic increased expression of messenger RNAs for erythropoietin protein or receptor, which in turn may lead to faster tumor growth, enhanced angiogenesis, and faster lymph node spread, ultimately resulting in tumor recurrence [30]. The progression and enhanced aggressiveness of endometrial cancer may be facilitated by hypoxia-inducible autocrine erythropoietin signaling. In endometrial carcinomas, elevated erythropoietin expression might be a stand-alone predictive or prognostic feature [31].

Further, in addition to the study of anemia in cancers, research on a link between ABO antigens and malignancies has increased in recent decades. It has recently been found that the presence of ABO antigens on the surface of cancer cells, despite their differences from those of normal cells, is nevertheless linked to the ability of these cells to evade an immune response and undergo apoptosis [32,33]. For example, Blood antigen A is linked to a higher risk of cancer of the oral cavity, whereas blood group B is linked to an increased incidence of hypopharyngeal cancer [32]. In the case of endometrial cancer, the positive connection of blood type A with cancer risk was seen regardless of menopausal state, body mass index, oral contraceptive usage, or family history of cancer [34]. The presence of anemia has an impact not only on the quality of life, but also on the long-term outcomes of cancer patients. Several studies have shown anemia to be a negative prognostic factor in multiple type of solid and hematological cancers. In endometrial cancer, the correlation between anemia at the time of diagnosis and patient evolution, response to treatment and survival has not been extensively researched. The prevalence of anemia has been reported between 18% and 42.6%, 8–10 recording a value of 38.26% in the present study. Tamussino et al. has shown an association between anemia and poor prognostic factors: more advanced FIGO stage, a higher rate of non-endometrioid histologic type, G2 and G3 tumor grading, adnexal involvement, lymph-vascular space invasion [14]. In another study, Metindir et al. suggests that the low pretreatment value of hemoglobin may reflect other unfavorable factors such as positive cytology and cervical involvement [12]. Anemia has been correlated with a negative impact on 5-year OS and 5 year disease-free, as shown by Wilairat et al. [13]. In these studies, anemia was proven only in univariate analysis to be a negative prognostic factor for endometrial cancer patients. In our series, we have found that along with age, LN metastasis and FIGO stage, a low pretreatment hemoglobin value is predictive of a negative long-term patient outcome, in both univariate and multivariate analysis. However, Younes et al. show that preoperative anemia was not significantly associated with a lower 5-year OS rate among patients with uterine papillary serous carcinoma [16].

Over the last decade, the approach to endometrial carcinoma has been transformed by the molecular stratification described by The Cancer Genome Atlas (TCGA) Research Network [35,36]. Surrogate testing revealed four molecular prognosis groups: p53 aberrant, POLE-mutated, MMR-deficient, and “no specific molecular profile” (NSMP) [36,37]. When the molecular categorization is applied to high-grade and/or high-risk endometrial carcinomas, it reveals that individuals with POLEmut tumors, which have a great prognosis, and those with p53-abnormal (p53abn) tumors, which have a bad prognosis, have different outcomes. Endometrial carcinomas having MMRd or non-specific molecular profile (NSMP) show an intermediate prognosis [37]. The endometrial cancer molecular prognostic categories based on TCGA have been incorporated into the ESGO-ESTRO-ESP guidelines [37]. Thus, ESGO/ESTRO/ESP recommendations divide the prognosis of endometrial cancer patients integrating TCGA genetic signature as well as pathological variables, such as lymphovascular space invasion [38]. Nevertheless, lymphovascular space invasion’s prognostic value is unrelated to the TCGA signature [38].

In the long-term evolution of endometrial cancer patients after initial treatment, relapse of disease may significantly reduce both survival and quality of life. Recurrence rates are generally low, having been reported in up to 15% of cases [8], in this study being 12.89%. In a study on a group of 424 patients with stage I endometrial cancer patients, Dunn et al., report 30 patients being diagnosed with relapse of disease [39]. Similarly, in a review of 923 patients with stages II to IV endometrial cancer, 91 cases were found with recurrence [40]. Relapse can be seen as either local or distant metastasis, but recurrences at multiple sites are not uncommon. Lymph node, vaginal, peritoneal and lung recurrence have been reported as the most frequent sites of relapse by Sohaib et al. [7]. Outcome of these patients depends on various factors. Sorbe et al. have proposed a preoperative and postoperative assessment of three tumor factors (histopathological type, FIGO stage and DNA ploidy) for the definition of patient risk groups [9]. Other studies have shown a strong correlation between type and localization of recurrence and patient long-term prognosis, suggesting that metastasis at multiple sites, distant compared to local recurrences and liver and spleen metastasis are negative prognostic factors [7,40,41]. Along with these factors TTR has been shown to have a significant correlation with OS rates, early relapse being associated with a poor prognosis [7]. Sorbe et al. reports a 2.1% per month decrease in mortality with TTR increasing [8]. Similarly, in a retrospective study Robbins et al., associates lower OS and disease-specific survival with a shorter TTR [41]. Mean TTR has been reported between 19.4 months and up to 32 months [8,41,42]. Anemia has not been found to predict or to be correlated with the occurrence of relapse of disease, but rather to have an influence on the TTR. In this study, we recorded a mean TTR of 7 months for patients with pretreatment anemia, compared to a mean interval of 14 months for the group of patients without low hemoglobin level. Although it has not been proven to be statistically significant, it can be suggested that the presence of anemia may shorten the time interval to recurrence in endometrial cancer patients.

Treatment options for metastatic disease include, but are not limited to, adjuvant radiotherapy or chemotherapy and surgery [8,11]. Whether curative treatment can be achieved in these cases depends on a series of factors, starting with type and location of recurrence and ending with comorbidities and nonetheless patients’ choice of therapy. Initiation of adjuvant therapy for recurrent disease may prove to impact patient survival, as has been suggested by Cattaneo et al. in their study, where the delay of adjuvant radiotherapy has been associated with a decrease in OS [43]. Regardless of type of treatment, curative or palliative, the endpoint is prolonging survival and maintaining a good quality of life.

This study has several limitations that warrant consideration. The retrospective cohort design introduces inherent biases, limiting the establishment of causal relationships. Single-center data collection from electronic medical records may result in variations in data quality and completeness. The exclusion of detailed information on specific post-surgical treatments and interventions represents a potential source of bias, as these factors may influence recurrence outcomes. Unmeasured confounders, such as socioeconomic factors or comorbidities, could contribute to residual bias. Moreover, a significant constraint in the present study is the absence of molecular classification data, a pivotal aspect that was not accessible within the clinical setting where the investigation was carried out. Also, another limitation of the study is the lack of important variables in the multivariate analysis, such as histological endometrioid grade and positive peritoneal cytology.

The interpretation of the results demands caution, acknowledging the study’s objectives and inherent limitations. The robust association between pre-operative anemia and adverse outcomes suggests the clinical relevance of anemia in endometrial cancer prognosis. The hazard ratios for anemia, age, FIGO stage, and lymph node metastasis emphasize their independent contributions to overall survival and early recurrence.

In light of our findings, there arises a promising avenue for future research focusing on the impact of pre-surgical anemia correction on surgical complications and prognosis of recurrence in endometrial cancer patients.

## 5. Conclusions

In conclusion, this study establishes pre-operative anemia as a significant predictor for both early recurrence and reduced overall survival in endometrial cancer patients. The findings underscore the clinical relevance of assessing and addressing anemia as part of the comprehensive management of these patients. Further prospective, multicenter investigations are imperative to validate these results and guide the development of targeted interventions aimed at improving outcomes for individuals with endometrial cancer and pre-operative anemia.

## Figures and Tables

**Table 1:** Clinical Characteristics of patients with endometrial cancer before surgery.

Variable	Total (*N* = 473)	Anemia (181/38.26%)	No Anemia (292/61.73%)	*p*-Value
Age	58 (11)	58 (12)	57 (10)	0.24
**HP type**				
Endometrioid adenocarcinoma	392/82.87%	144/79.55%	248/84.93%	0.13
Others	81/17.12%	37/20.44	44/15.06%	
**FIGO stage**				
IA	5/1.05%	-	5/1.71%	-
IB	13/2.74%	3/1.65%	10/3.42%	0.38
II	202/42.70%	49/27.07%	153/52.39%	<0.0001
IIIA	30/6.34%	17/9.39%	13/4.45%	0.05
IIIB	174/36.78%	85/46.96%	89/30.47%	0.0004
IIIC1	23/4.86%	12/6.62%	11/3.76%	0.18
IIIC2	14/2.95%	7/3.86%	7/2.39%	0.40
IVA	12/2.53%	8/4.41%	4/1.36%	0.06
**Myometrial invasion >50%**	410/86.49%	177/97.79	233/79.79%	<0.0001
**Surgery type**				
HTL	461/97.46%	173/95.58%	288/98.63%	0.06
PLV	12/2.53%	8/4.41%	4/1.36%	
**Lymph node metastases**	49/10.35%	27/14.91%	22/7.53%	0.01
**Adnexal involvement**	146/30.86%	75/41.43%	71/24.31%	0.0001
**Cervical involvement**	316/66.80%	128/70.71%	188/64.38%	0.16
**Lymphovascular invasion**	433/91.54%	179/98.89%	254/86.98%	<0.0001
**Hb (g/dL)**	12.3 (3.09)	12.9 (1.3)	9.3 (1.9)	
**Recurrence**	61/12.89%	34/18.78%	27/9.24%	0.004

Note: Data for continuous variables (Age and Hb) are presented as median and interquartile range (IQR). Hb = Hemoglobin level; HTL = Total hysterectomy and bilateral adnexectomy with pelvic lymphadenectomy; PLV = anterior/posterior/total pelvectomy.

**Table 2:** Cox regression analysis of the relationship anemia, age, FIGO stage, LN metastasis, myometrial invasion > 50%, non-endometroid histology and cancer mortality.

Variable	Coef.	HR	SE (Coef.)	*p*-Value
Anemia—Yes	0.31	1.36	0.14	0.02
Age	0.08	1.08	0.009	<0.0001
FIGO II	0.10	1.09	0.594	0.86
FIGO III	1.40	4.06	0.601	0.01
FIGO IV	2.94	18.94	0.717	<0.0001
LN metastasis-Yes	0.71	2.03	0.227	0.001
Myometrial invasion > 50%	0.04	1.04	0.296	0.87
Non-endometroid histology	0.40	1.50	0.168	0.01

Note: coef = regression coefficients.

**Table 3:** Univariate and multivariate binomial logistic regression analysis between presence of anemia and mortality.

Variable	Estimate	SE	OR	*p*-Value
Univariate analysis				
Anemia—Yes	0.762	0.192	2.14	<0.0001
Multivariate analysis				
Anemia—Yes	0.474	0.236	1.60	0.04
Age	0.117	0.016	1.12	<0.001
FIGO II	−0.351	0.697	0.703	0.61
FIGO III	0.519	0.704	1.68	0.46
FIGO IV	15.78	6.05	715	0.97
LN metastasis-Yes	0.676	0.418	1.96	0.10
Myometrial invasion > 50%	0.118	0.405	1.12	0.77
Non-endometroid histology	0.815	0.349	2.26	0.01

**Table 4:** Univariate and multivariate binomial logistic regression analysis between presence of anemia and risk of recurrence.

Variable	Estimate	SE	OR	*p*-Value
Univariate analysis				
Anemia—Yes	0.819	0.277	2.27	0.003
Multivariate analysis				
Anemia—Yes	0.749	0.298	2.11	0.01
Age	0.004	0.017	1.004	0.80
FIGO II	−0.659	0.703	0.516	0.34
FIGO III	−0.741	0.723	0.476	0.30
FIGO IV	0.293	1.040	1.34	0.77
LN metastasis-Yes	0.513	0.476	1.67	0.28
Myometrial invasion > 50%	0.242	0.540	1.27	0.65
Non-endometroid histology	−0.078	0.400	0.92	0.84

Outcome = early recurrence.

**Table 5:** Cox regression analysis of the relationship anemia, age, FIGO stage, LN metastasis and cancer recurrence.

Variable	Coef.	HR	SE (Coef.)	*p*-Value
Anemia—Yes	2.454	11.64	0.401	<0.0001
Age	0.043	1.044	0.023	0.006
FIGO II	0.595	1.813	0.716	0.40
FIGO III	0.448	1.565	0.734	0.54
FIGO IV	0.487	1.628	0.958	0.61
LN metastasis-Yes	0.864	2.374	0.488	0.04
Myometrial invasion > 50%	0.621	1.862	0.627	0.32
Non-endometroid histology	0.809	2.246	0.399	0.04

Note: coef = regression coefficients.

## Data Availability

The data sets used and/or analyzed during the present study are available from the first author on reasonable request.

## References

[1] Sung H., Ferlay J., Siegel R.L., Laversanne M., Soerjomataram I., Jemal A., Bray F. (2021). Global Cancer Statistics 2020: GLOBOCAN Estimates of Incidence and Mortality Worldwide for 36 Cancers in 185 Countries. CA Cancer J. Clin..

[2] Mahdy H., Casey M.J., Crotzer D. (2023). Endometrial Cancer. StatPearls.

[3] Rahib L., Smith B.D., Aizenberg R., Rosenzweig A.B., Fleshman J.M., Matrisian L.M. (2014). Projecting Cancer Incidence and Deaths to 2030: The Unexpected Burden of Thyroid, Liver, and Pancreas Cancers in the United States. Cancer Res..

[4] Endometrial Cancer Statistics|World Cancer Research Fund International. https://www.wcrf.org/cancer-trends/endometrial-cancer-statistics/.

[5] Key Statistics for Endometrial Cancer. https://www.cancer.org/cancer/types/endometrial-cancer/about/key-statistics.html.

[6] Sant M., Chirlaque Lopez M.D., Agresti R., Sánchez Pérez M.J., Holleczek B., Bielska-Lasota M., Dimitrova N., Innos K., Katalinic A., Langseth H. (2015). Survival of Women with Cancers of Breast and Genital Organs in Europe 1999–2007: Results of the EUROCARE-5 Study. Eur. J. Cancer.

[7] Sohaib S.A., Houghton S.L., Meroni R., Rockall A.G., Blake P., Reznek R.H. (2007). Recurrent Endometrial Cancer: Patterns of Recurrent Disease and Assessment of Prognosis. Clin. Radiol..

[8] Sorbe B., Juresta C., Ahlin C. (2014). Natural History of Recurrences in Endometrial Carcinoma. Oncol. Lett..

[9] Sorbe B. (2012). Predictive and Prognostic Factors in Definition of Risk Groups in Endometrial Carcinoma. ISRN Obstet. Gynecol..

[10] Bosse T., Peters E.E.M., Creutzberg C.L., Jürgenliemk-Schulz I.M., Jobsen J.J., Mens J.W.M., Lutgens L.C.H.W., Van Der Steen-Banasik E.M., Smit V.T.H.B.M., Nout R.A. (2015). Substantial Lymph-Vascular Space Invasion (LVSI) Is a Significant Risk Factor for Recurrence in Endometrial Cancer—A Pooled Analysis of PORTEC 1 and 2 Trials. Eur. J. Cancer.

[11] Huijgens A.N.J., Mertens H.J.M.M. (2013). Factors Predicting Recurrent Endometrial Cancer. Facts Views Vis. Obgyn.

[12] Metindir J., Bilir Dilek G. (2009). Preoperative Hemoglobin and Platelet Count and Poor Prognostic Factors in Patients with Endometrial Carcinoma. J. Cancer Res. Clin. Oncol..

[13] Wilairat W., Benjapibal M. (2012). Presence of Anemia and Poor Prognostic Factors in Patients with Endometrial Carcinoma. Asian Pac. J. Cancer Prev..

[14] Tamussino K.F., Gucer F., Reich O., Moser F., Petru E., Scholz H.S. (2001). Pretreatment Hemoglobin, Platelet Count, and Prognosis in Endometrial Carcinoma. Int. J. Gynecol. Cancer.

[15] Njølstad T.S., Engerud H., Werner H.M.J., Salvesen H.B., Trovik J. (2013). Preoperative Anemia, Leukocytosis and Thrombocytosis Identify Aggressive Endometrial Carcinomas. Gynecol. Oncol..

[16] Younes G., Segev Y., Begal J., Auslender R., Goldberg Y., Amit A., Lavie O. (2016). The Prognostic Significance of Hematological Parameters in Women with Uterine Serous Papillary Carcinoma (USPC). Eur. J. Obstet. Gynecol. Reprod. Biol..

[17] Abu-Zaid A., Alomar O., Abuzaid M., Baradwan S., Salem H., Al-Badawi I.A. (2021). Preoperative Anemia Predicts Poor Prognosis in Patients with Endometrial Cancer: A Systematic Review and Meta-Analysis. Eur. J. Obstet. Gynecol. Reprod. Biol..

[18] Biete A., Holub K. (2017). Haemoglobin Monitoring in Endometrial Cancer Patients Undergoing Radiotherapy. Clin. Transl. Oncol..

[19] Becker P.S., Griffiths E.A., Alwan L.M., Bachiashvili K., Brown A., Cool R., Curtin P., Dinner S., Gojo I., Hicks A. (2020). NCCN Guidelines Insights: Hematopoietic Growth Factors, Version 1.2020. J. Natl. Compr. Cancer Netw..

[20] Rodgers G.M., Becker P.S., Blinder M., Cella D., Chanan-Khan A., Cleeland C., Coccia P.F., Djulbegovic B., Gilreath J.A., Kraut E.H. (2012). Cancer- and Chemotherapy-Induced Anemia. J. Natl. Compr. Cancer Netw..

[21] Aapro M., Beguin Y., Bokemeyer C., Dicato M., Gascón P., Glaspy J., Hofmann A., Link H., Littlewood T., Ludwig H. (2018). Management of Anaemia and Iron Deficiency in Patients with Cancer: ESMO Clinical Practice Guidelines. Ann. Oncol..

[22] Anic K., Schmidt M.W., Schmidt M., Krajnak S., Löwe A., Linz V.C., Schwab R., Weikel W., Brenner W., Westphalen C. (2022). Impact of Perioperative Red Blood Cell Transfusion, Anemia of Cancer and Global Health Status on the Prognosis of Elderly Patients with Endometrial and Ovarian Cancer. Front. Oncol..

[23] El Ghouayel M., Hamidi M., Mazis C., Imam Z., Abbad M., Boutall A., Guerrero M., Nfonsam V. (2022). Surgical Outcomes in Patients with Preoperative Anemia Undergoing Colectomy for Colon Cancer. J. Surg. Res..

[24] Siegel R.L., Miller K.D., Jemal A. (2015). Cancer Statistics, 2015. CA Cancer J. Clin..

[25] Ferlay J., Soerjomataram I., Dikshit R., Eser S., Mathers C., Rebelo M., Parkin D.M., Forman D., Bray F. (2015). Cancer Incidence and Mortality Worldwide: Sources, Methods and Major Patterns in GLOBOCAN 2012. Int. J. Cancer.

[26] Van Belle S.J.-P. (2004). What Is the Value of Hemoglobin as a Prognostic and Predictive Factor in Cancer?. Eur. J. Cancer Suppl..

[27] Rochet N.M., Markovic S.N., Porrata L.F. (2012). The Role of Complete Blood Cell Count in Prognosis—Watch This Space!. Oncol. Hematol. Rev..

[28] Madeddu C., Gramignano G., Astara G., Demontis R., Sanna E., Atzeni V., Macciò A. (2018). Pathogenesis and Treatment Options of Cancer Related Anemia: Perspective for a Targeted Mechanism-Based Approach. Front. Physiol..

[29] Adamson J.W. (2008). The Anemia of Inflammation/Malignancy: Mechanisms and Management. Hematology.

[30] Yasuda Y. (2002). Erythropoietin Is Involved in Growth and Angiogenesis in Malignant Tumours of Female Reproductive Organs. Carcinogenesis.

[31] Acs G., Xu X., Chu C., Acs P., Verma A. (2004). Prognostic Significance of Erythropoietin Expression in Human Endometrial Carcinoma. Cancer.

[32] Alexandra G., Alexandru M., Stefan C.F., Petruta-Maria D., Gabriel B.M., Dragos-Eugen G., Teodor G.M. (2022). Blood Group Type Association with Head and Neck Cancer. Hematol. Rep..

[33] Huang J.Y., Wang R., Gao Y.-T., Yuan J.-M. (2017). ABO Blood Type and the Risk of Cancer—Findings from the Shanghai Cohort Study. PLoS ONE.

[34] Xu W.-H., Zheng W., Xiang Y.-B., Shu X.-O. (2011). ABO Blood Type Is Associated with Endometrial Cancer Risk in Chinese Women. Chin. J. Cancer.

[35] Levine D.A., The Cancer Genome Atlas Research Network (2013). Integrated Genomic Characterization of Endometrial Carcinoma. Nature.

[36] Arciuolo D., Travaglino A., Raffone A., Raimondo D., Santoro A., Russo D., Varricchio S., Casadio P., Inzani F., Seracchioli R. (2022). TCGA Molecular Prognostic Groups of Endometrial Carcinoma: Current Knowledge and Future Perspectives. IJMS.

[37] Concin N., Matias-Guiu X., Vergote I., Cibula D., Mirza M.R., Marnitz S., Ledermann J., Bosse T., Chargari C., Fagotti A. (2021). ESGO/ESTRO/ESP Guidelines for the Management of Patients with Endometrial Carcinoma. Int. J. Gynecol. Cancer.

[38] Raffone A., Travaglino A., Raimondo D., Neola D., Maletta M., Santoro A., Insabato L., Casadio P., Fanfani F., Zannoni G.F. (2022). Lymphovascular Space Invasion in Endometrial Carcinoma: A Prognostic Factor Independent from Molecular Signature. Gynecol. Oncol..

[39] Dunn E.F., Geye H., Platta C.S., Gondi V., Rose S., Bradley K.A., Anderson B.M. (2014). Predictive Factors of Recurrence Following Adjuvant Vaginal Cuff Brachytherapy Alone for Stage I Endometrial Cancer. Gynecol. Oncol..

[40] Huang H.-J., Tang Y.-H., Chou H.-H., Yang L.-Y., Chao A., Huang Y.-T., Lin G., Liu F.-Y., Chang T.-C., Lai C.-H. (2014). Treatment Failure in Endometrial Carcinoma. Int. J. Gynecol. Cancer.

[41] Robbins J.R., Yechieli R., Laser B., Mahan M., Rasool N., Elshaikh M.A. (2012). Is Time to Recurrence after Hysterectomy Predictive of Survival in Patients with Early Stage Endometrial Carcinoma?. Gynecol. Oncol..

[42] Bell J.G., Patterson D.M., Klima J., Harvison M., Rath K., Reid G. (2014). Outcomes of Patients with Low-Risk Endometrial Cancer Surgically Staged without Lymphadenectomy Based on Intra-Operative Evaluation. Gynecol. Oncol..

[43] Cattaneo R., Hanna R.K., Jacobsen G., Elshaikh M.A. (2014). Interval Between Hysterectomy and Start of Radiation Treatment Is Predictive of Recurrence in Patients with Endometrial Carcinoma. Int. J. Radiat. Oncol. Biol. Phys..

